# Automated PD-L1 Scoring for Non-Small Cell Lung Carcinoma Using Open-Source Software

**DOI:** 10.3389/pore.2021.609717

**Published:** 2021-03-26

**Authors:** Julia R. Naso, Tetiana Povshedna, Gang Wang, Norbert Banyi, Calum MacAulay, Diana N. Ionescu, Chen Zhou

**Affiliations:** ^1^ Department of Pathology and Laboratory Medicine, University of British Columbia, Vancouver, BC, Canada; ^2^ Department of Pathology, BC Cancer, Vancouver, BC, Canada; ^3^ Department of Integrative Oncology, British Columbia Cancer Research Center, Vancouver, BC, Canada

**Keywords:** pathology, PD-L1, biomarker, non-small cell lung cancer, digital pathology

## Abstract

PD-L1 expression in non-small cell lung cancer (NSCLC) is predictive of response to immunotherapy, but scoring of PD-L1 immunohistochemistry shows considerable interobserver variability. Automated methods may allow more consistent and expedient PD-L1 scoring. We aimed to assess the technical concordance of PD-L1 scores produced using free open source QuPath software with the manual scores of three pathologists. A classifier for PD-L1 scoring was trained using 30 NSCLC image patches. A separate test set of 207 image patches from 69 NSCLC resection cases was used for comparison of automated and manual scores. Automated and average manual scores showed excellent correlation (concordance correlation coeffecient = 0.925), though automated scoring resulted in significantly more 1–49% scores than manual scoring (*p* = 0.012). At both 1% and 50% thresholds, automated scores showed a level of concordance with our ‘gold standard’ (the average of three pathologists’ manual scores) similar to that of individual pathologists. Automated scoring showed high sensitivity (95%) but lower specificity (84%) at a 1% threshold, and excellent specificity (100%) but lower sensitivity (71%) at a 50% threshold. We conclude that our automated PD-L1 scoring system for NSCLC has an accuracy similar to that of individual pathologists. The detailed protocol we provide for free open source scoring software and our discussion of the limitations of this technology may facilitate more effective integration of automated scoring into clinical workflows.

## Introduction

The treatment of advanced-stage non-small cell lung cancer (NSCLC) has seen considerable advances with the introduction of immunotherapy [1]. Expression of programmed death ligand 1 (PD-L1) is predictive of response to treatment with PD-1 and PD-L1 inhibitors [2]. PD-L1 expression has conventionally been manually scored as the proportion of tumor cells with any membranous staining. Thresholds of 1% and 50% have been used for different inhibitors [3]. However, there exists considerable interobserver variability in PD-L1 scoring [4–6], a factor that can limit the predictive value of PD-L1 testing. There is also no gold standard for “true” PD-L1 scores, though technical concordance of results across multiple pathologists has supported the interchangeability of different PD-L1 assays [3, 7].

Automated scoring of digital slide images is a potential means of high-throughput precise and accurate PD-L1 scoring, which may reveal more robust associations with treatment response. However, performance is likely to differ between methodologies, many of which require locally unavailable or proprietary software. Studies using proprietary software or in-house developed algorithms have produced PD-L1 scores comparable to pathologists [8, 9], but implementation may be complicated by the need for funding and licensure agreements. Free open source software may therefore provide a more accessible option for automated scoring.

The free open source program QuPath is notable as it does not require any software expertize or coding skill to create custom scoring approaches [10, 11]. The application of QuPath to PD-L1 scoring was first reported in the setting of colorectal carcinoma [11]. The resulting PD-L1 scores had prognostic value, but a comparison with manual scoring was not provided. A subsequent study using QuPath to score NSCLC PD-L1 showed promising results [12]; However, it remains unclear whether the degree of deviation of QuPath automated scores from their “gold standard” is within the range of interobserver variability between individual pathologist’s manual scores. While detailed descriptions of the variables that can be customized in QuPath are available from its developers, the literature is lacking a simple step-wise protocol for the development and implementation of QuPath PD-L1 scoring in NSCLC. We aimed to provide such a protocol and demonstrate how the resulting automated scores compare to manual pathologist scores. We also analyze sources of discordance between automated and manual scoring and discuss how the limitations of automated scoring may affect the integration of this technology into clinical PD-L1 testing workflows.

## Methods

This study was approved by the University of British Columbia Research Ethics Board (H18–01619, approved Aug 27th, 2018). Cases were identified retrospectively from the British Columbia Cancer (BC Cancer) archives. PD-L1 immunohistochemistry was performed on freshly cut sections using 22C3 antibody (#M365-3, Dako/Agilent, Santa Clara, CA, United States) on the VENTANA BenchMark ULTRA IHC/ISH system (Ventana/Roche, Tucson, AZ, United States) following a protocol previously demonstrated to have analytical concordance with the commercial 22C3 PharmDx and VENTANA SP263 assays [13]. ULTRA cell conditioning solution (#950-224, Ventana/Roche, Tucson, AZ, United States) was applied for 48 min, followed by a 64 min room temperature incubation with 1:40 PD-L1 antibody and detection using the OptiView DAB IHC Detection Kit (#760-700, Ventana/Roche, Tucson, AZ, United States). Slides were immunostained in multiple batches interspersed with clinical cases.

Cases were scanned on a MoticEasyScan Infinity instrument (Motic Digital Pathology, Richmond, BC, Canada) at x40 magnification. Three image patches from each case were selected by a pathologist to represent different tumor morphologies and PD-L1 staining levels, and were exported as tiff images for scoring. Image patch size in testing and training sets is indicated in Table 1. Test set image patches were at most 4.0 mm^2^, allowing pathologists to focus on detailed examination and precise scoring of a small area, but included a range of sizes to assess whether automated scoring could perform robustly over a size range.

**TABLE 1:** Case demographics.

Category	Training set		Testing set	
	n	%	n	%
Sex				
Female	5	50%	35	51%
Male	5	50%	34	49%
Diagnosis				
Adenocarcinoma, non-mucinous	8	80%	58	84%
Squamous cell carcinoma	1	10%	11	16%
Non-small cell carcinoma NOS	1	10%	0	0%
Site				
Lung	10	100%	66	94%
Metastasis to bone	0	0%	1	1%
Metastasis to brain	0	0%	2	3%
Procedure				
Lobectomy	6	60%	45	65%
Wedge resection	4	40%	21	30%
Large resection of metastatic tumor	0	0%	3	4%
Image patch area [mm^2^, median (range)]	0.94 [0.16–7.94]	NA	0.44 [0.05–4.01]	NA

Automated scoring of each image patch used QuPath software (version 0.1.2) [10, 11]. A detailed step-by-step protocol is provided in Supplementary Material. Briefly, the stains were separated using color deconvolution, and nuclei were identified based on user-specified morphological parameters in the hematoxylin channel. Cell areas were estimated based on the proximity of neighboring nuclei and specified parameters. Sixty-seven morphological features calculated for each cell were used as input for a random trees classifier. The classifier was trained to distinguish tumor cells from background cell populations through user annotation of tumor regions in 30 image patches from 10 randomly selected cases. Classifier outputs were displayed as image mark-ups throughout the process of annotation, allowing continual monitoring of classifier performance. Automated PD-L1 scores were calculated as the percent of tumor cells whose mean DAB optical density exceeded an empirically determined threshold.

Manual scores for the digital image patches were obtained independently from three pathologists with training and experience in PD-L1 scoring (G.W., C.Z. and D.N.I.), blinded to the clinically reported PD-L1 score. Membranous staining of any intensity in tumor cells was counted as positive, and expressed as a percentage of the total number of tumor cells on a continuous scale. Average manual scores were calculated by first averaging the continuous-scale scores of individual pathologists, then placing the average continuous scores into categories (<1%, 1–49% and ≥50%). Case level QuPath and pathologist scores were calculated as the weighted average of the continuous-scale scores on the three image patches, weighted according to what proportion of the total number of tumor cells (according to QuPath) were in each image. Thus, the case level QuPath scores are equal to the total number of ‘positive’ cells across all three image patches, divided by the total number of tumor cells across all three image patches. The resulting continuous-scale case level scores were then placed in <1%, 1–49% and ≥50% categories.

Statistical analysis was performed using the R Project for Statistical Computing (version 3.5.2) in RStudio version 1.2.1335. Continuous scores were compared using Lin’s concordance correlation coefficient and Wilcoxon signed rank tests (for paired data) or Mann-Whitney *U*-tests (for independent data). Categorical scores were compared using a Chi-squared test. *p*-values < 0.05 were considered statistically significant. Agreement between scoring methods was assessed using Cohen’s kappa, with kappa values interpreted as follows: 0.40–0.69 indicates weak agreement, 0.70–0.79 indicates moderate agreement, 0.80–0.89 indicates strong agreement and ≥0.9 indicates near perfect agreement) [3].

## Results

**FIGURE 1:**
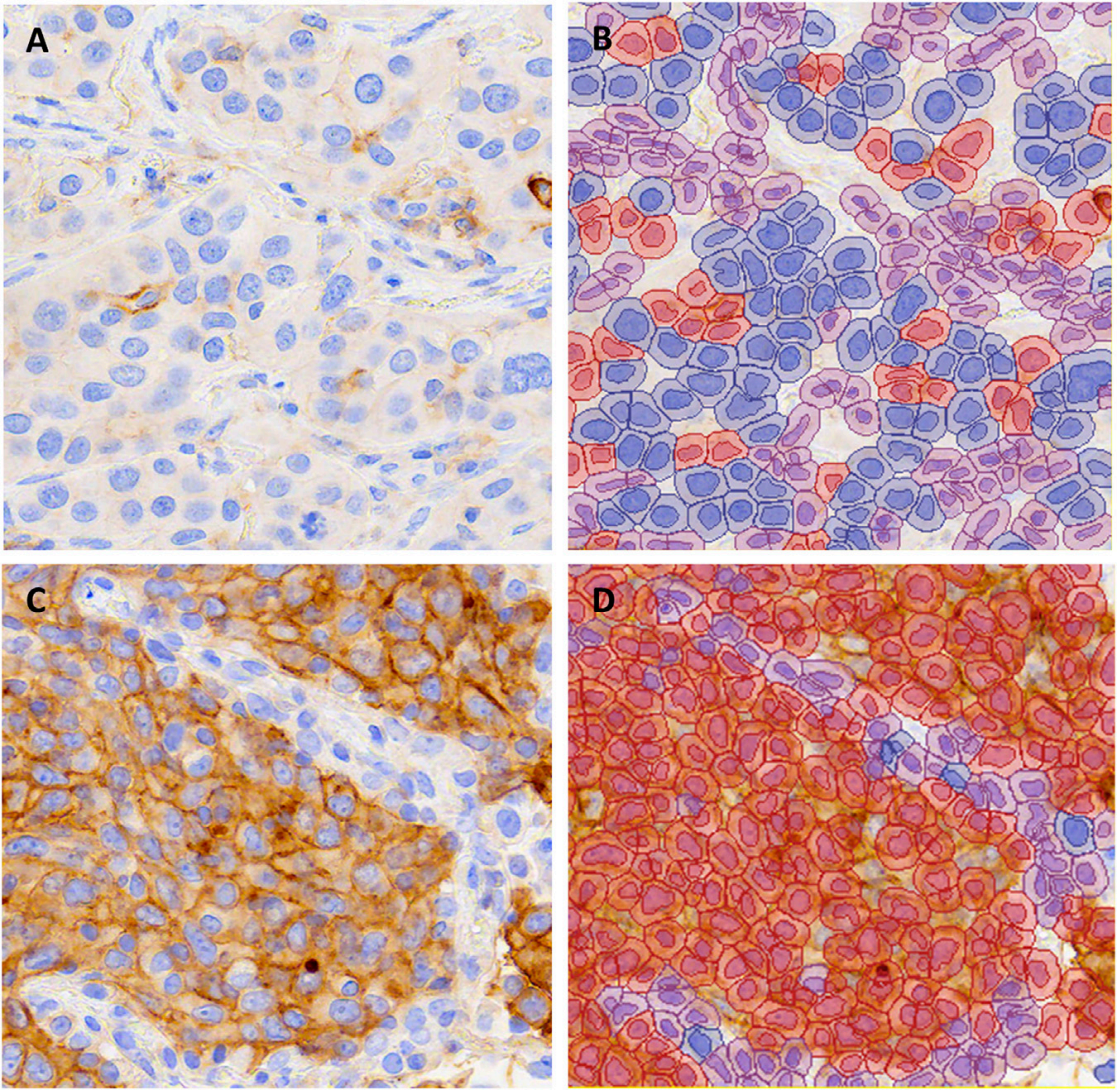
Representative images of PD-L1 stained non-small cell lung carcinomas from **(A,B)** a case with TPS 1–49% and **(C,D)** a case with TPS ≥50% in all scoring methods (∼X200). The unannotated image is shown in **(A,C)** and QuPath annotations are shown in **(B,D)**. Blue indicates PD-L1 negative tumor cells, red indicates PD-L1 positive tumor cells and purple indicates background stroma and immune cells.

Three image patches representative of different tumor morphologies and PD-L1 staining levels were selected for each of 79 large resection cases of NSCLC (case demographics in Table 1). The image patches from 10 randomly selected cases were used for training the QuPath classifier (*n* = 30 image patches), while image patches from the remaining 69 cases (*n* = 207 image patches) were used for testing the performance of automated scoring. The independent manual scores from three pathologists were compared to automated scores for the same patches (representative images in Figure 1). Analysis of the 207 images patches was calculated to provide adequate power (i.e., power ≥0.80) for detecting a difference in kappa of 0.11 (assuming a standard deviation of 0.4 and alpha of 0.05).

When using a continuous scale, automated scores were strongly correlated with the average of the three pathologist’s manual scores (Lin’s concordance correlation coefficient 0.925 (95% confidence interval 0.903–0.942). When converted to categorical scores, there was concordance between automated and average pathologist scores for 186/207 images (90%) when using a 1% positivity threshold, and 191/207 images (92%) when using a 50% positivity threshold. Similar rates of concordance were seen between individual pathologist’s scores and the average manual scores (91–95% concordant when using 1% positivity threshold, and 95–97% concordant when using 50% positivity threshold, Table 2). Cohen’s kappa statistic for agreement between automated and average manual scores (κ = 0.80 for 1% threshold; κ = 0.78 for 50% threshold) was not significantly different from the kappa statistics for agreement between each individual pathologist and the average manual score (Table 2). Interobserver variability in pairwise comparisons between the individual pathologists (1% threshold: average 88% concordance, average κ = 0.76; 50% threshold: average 93% concordance, average κ = 0.82) was similar to estimates of interobserver variability in the literature [6, 14, 15].

**TABLE 2:** Agreement of automated scores and individual pathologist’s manual scores with the average manual score for single image patches.

Threshold	Scoring method	Concordant with average manual score (n [%])	Cohen's kappa for agreement with average manual score (95% CI)	Sensitivity^a^ (%)	Specificity^a^ (%)
1	QuPath	186/207 (90%)	0.80 (0.71–0.88)	95	84
	Pathologist #1	188/207 (91%)	0.82 (0.74–0.89)	83	100
	Pathologist #2	191/207 (92%)	0.85 (0.77–0.92)	87	98
	Pathologist #3	197/207 (95%)	0.90 (0.84–0.96)	98	92
50	QuPath	191/207 (92%)	0.78 (0.69–0.92)	71	100
	Pathologist #1	200/207 (97%)	0.91 (0.85–0.98)	95	97
	Pathologist #2	196/207 (95%)	0.87 (0.80–0.95)	98	93
	Pathologist #3	201/207 (97%)	0.93 (0.87–0.98)	93	99

^a^ The average manual score was used as the ‘gold standard’ for sensitivity and specificity calculations.

**FIGURE 2:**
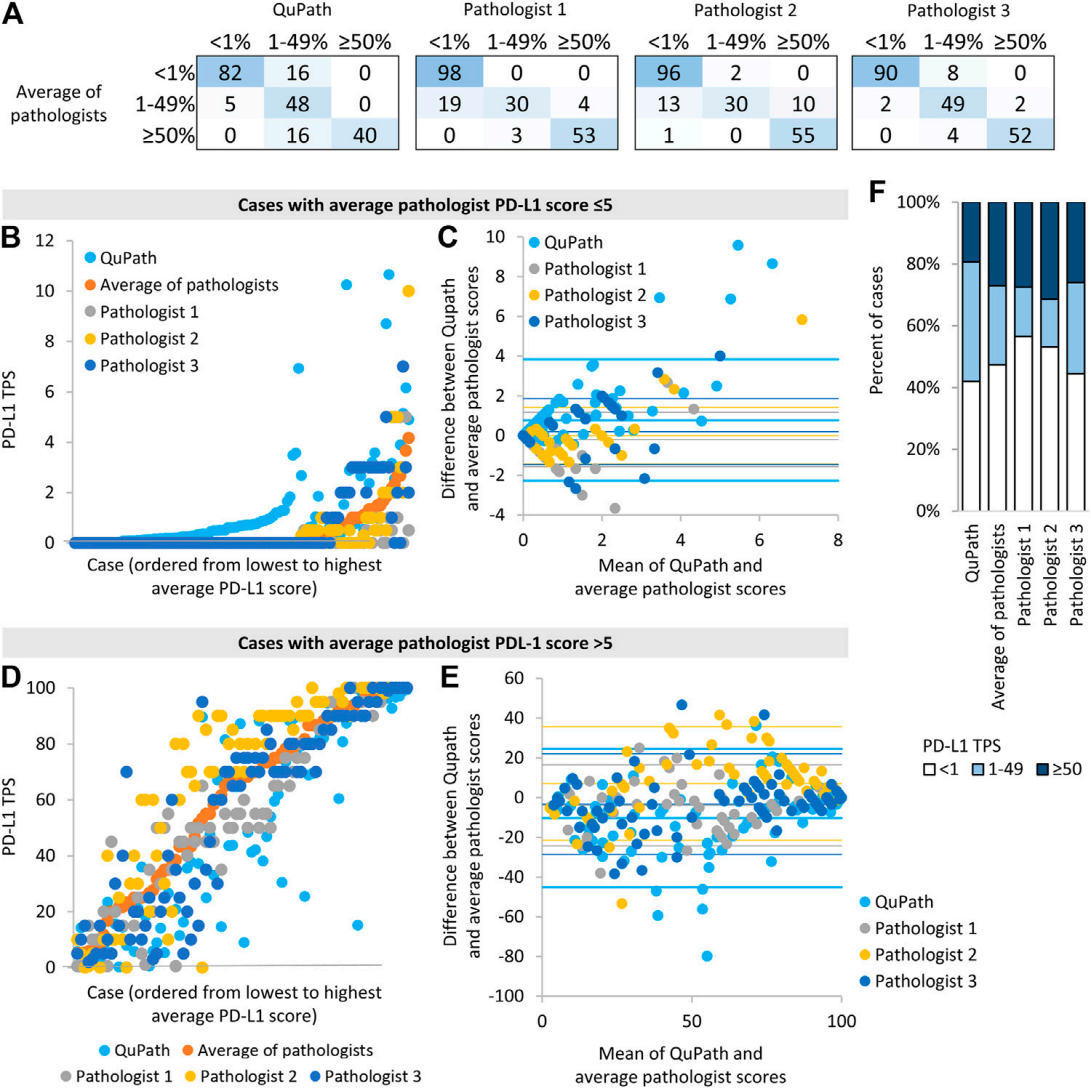
Comparison of automated (“QuPath”) and manual (“pathologist”) PD-L1 scores **(A)** The number of image patches in each PD-L1 TPS category when using scores from automated or single pathologist scoring compared to average pathologist scores **(B,D)** Scores for each image patch ordered by the average pathologist score **(C,E)** Bland-Altman plots of the difference in scores vs. the mean of scores for different scoring methods compared to average pathologist scores. For each data series, the horizontal lines represent two standard deviation above the mean difference, the mean difference, and two standard deviations below the mean difference. Data for image patches and data for image patches with an average score ≤5% are shown in **(B,C)**, and those with an average pathologist score >5% are shown in **(D,E)**. **(D)** The proportion of image patches in each PD-L1 score category according to each scoring method.

We then assessed whether automated scoring tended to increase or decrease scores relative to the average pathologist score. Cases discordant across the 1% threshold most often had higher scores in automated than manual analysis (i.e., 16 image patches had 1–49% QuPath scores and <1% average pathologist scores, whereas only 5 image patches had <1% QuPath scores and 1–49% average pathologist scores, Figure 2A). When evaluated on a continuous scale, automated scores were on average 0.8% higher than average pathologist scores, for cases with average manual scores ≤5% (*p* < 0.001, Figures 2B,C). When using a 1% threshold for positivity and considering the average manual score to be the gold standard, automated scoring had a high sensitivity for positive scores (95%), similar to individual pathologists, but had a lower specificity (84%) than individual pathologists (Table 2).

In contrast, cases discordant around the 50% threshold all scored lower in automated than manual analysis (i.e., all 16 discordant cases had 1–49% QuPath scores and ≥50% average pathologist scores). When evaluated on a continuous scale, automated scores were on average 10% lower than average pathologist scores, for cases with average pathologist scores >5% (*p* < 0.001, Figures 2D,E). The continuous scores from individual pathologists also deviated significantly from the average manual scores (e.g., one pathologist’s scores were on average 7% higher than average pathologists scores, *p* < 0.001 Figures 2D,E), in keeping with the notion that automated and individual pathologist scores have similar accuracy relative to the ‘gold standard’ average manual score. When using a 50% threshold for positivity and considering the average manual score to be the gold standard, automated scoring had an excellent specificity for positive scores (100%) but had a lower sensitivity (71%) than individual pathologists (Table 2).

Reflecting the tendency of automated scoring to underestimate the PD-L1 score of high-scoring cases and overestimate the PD-L1 score of low-scoring cases, more image patches scored in the 1–49% category when using automated rather than average manual scores (*p* = 0.012, Figure 2F). Images with an automated score of 1–49% were most likely to be discordant with the average manual score: There was 40% discordance for images with an automated score of 1–49%, 6% discordance for images with an automated score <1, and 0% discordance for images with an automated score ≥50%. There was no significant association between discordance and adenocarcinoma vs. squamous cell carcinoma diagnosis (*p* = 0.96), image patch area (*p* = 0.96), the number of tumor cells identified by Qupath (*p* = 0.058) or primary vs. metastatic site sampling (*p* = 0.29).

**FIGURE 3:**
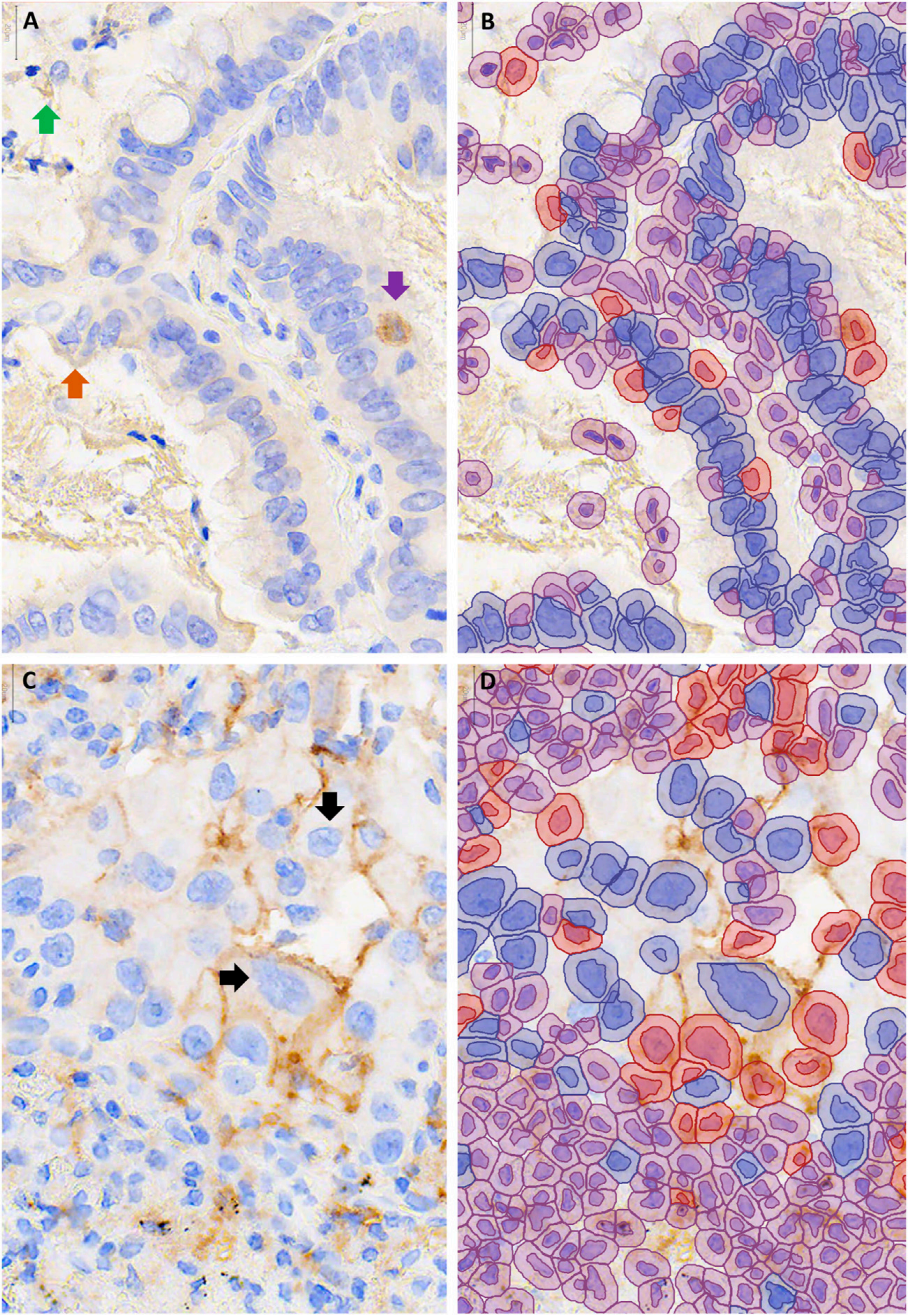
Examples of cases with discordant automated and manual PD-L1 scores. QuPath annotations **(B,D)** are shown to the right of the corresponding unannotated PD-L1 stained area, 400–600X magnification **(A,C)**. **(A,B)** Over-estimation of PD-L1 staining using QuPath was contributed to by false positive annotation of debris (green arrow), inflammatory cells (purple arrow) and edge artifact (orange arrow). **(C,D)** Under-estimation of PD-L1 staining using QuPath was contributed to by cell area being underestimated, such that membranous staining fell outside of the annotated area of the cell **↓**.

Examination of the 37 image patches with discordant automated and average manual scores revealed that 11 (30%) had at least one pathologist in agreement with the automated score category, such that the automated score could be viewed as ‘correct’ depending on the pathologist. Of the remaining 26 images, 13 scored higher by automated than manual scoring (i.e., 1-49% rather than <1%) but tended to have scores close to the threshold: 9 of those images scored <2% and 7 exceeded 1% threshold as a result of 4 or fewer cells being called positive. On review of the QuPath annotations, factors that contributed to overestimation of PD-L1 scores included debris miscalled as positive staining (in 6 images), positively staining stromal cells miscalled as tumor (in 6 images) and positively staining airspace macrophages miscalled as tumor cells (in 5 images, Figures 3A,B). Very faint brown staining at the edge of a tumor fragment (“edge-artifact” to the human eye) was called positive staining by QuPath in 2 images. In one case of squamous cell carcinoma, mis-designation of negative-staining tumor cells as stromal cells falsely elevated the PD-L1 score. The remaining 13 out 26 images with automated scores discordant across all pathologists had lower automated than pathologist scores. Of those, 11 had automated scores in the 1–49% category, including 7 that scored >40%. Factors that contributed to the underestimation of PD-L1 staining included very faint staining being miscalled as negative (in 7 images) and membrane staining falling outside of the area designated as tumor cell (in 8 images, Figures 3C,D).

To simulate diagnostic evaluation of a full case, we calculated weighted averages of the continuous-scale scores for the three image patches per case (see Methods) to produce single ‘case level’ scores, which we then placed in to <1%, 1–49% and ≥50% categories. Though the reduced sample size (*n* = 69 rather than 207) limited the power of this analysis (power = 0.6 for detecting a 0.15 difference in kappa, assuming a standard deviation of 0.4 and alpha of 0.05), we noted similar percent concordance, sensitivity and specificity (Table 3) as in the single patch analysis above, supporting that the trends discussed above are likely to hold true over larger tissue areas and potentially whole slides.

**TABLE 3:** Agreement of automated scores and individual pathologist’s manual scores with the average manual score for each patient case.

Threshold (%)	Scoring method	Concordant with average manual score (n [%])	Cohen’s kappa for agreement with average manual score (95% CI)	Sensitivity^a^ (%)	Specificity^a^ (%)
1	QuPath	64/69 (93%)	0.77 (0.62–0.93)	98	85
	Pathologist #1	63/69 (91%)	0.84 (0.71–0.97)	88	96
	Pathologist #2	67/69 (97%)	0.94 (0.86–1)	95	100
	Pathologist #3	65/69 (94%)	0.87 (0.76–0.99)	98	88
50	QuPath	63/69 (91%)	0.76 (0.58–0.94)	68	100
	Pathologist #1	68/69 (99%)	0.96 (0.89–1)	95	100
	Pathologist #2	67/69 (97%)	0.93 (0.83–1)	100	96
	Pathologist #3	67/69 (97%)	0.93 (0.83–1)	95	98

^a^ The average manual score was used as the ‘gold standard’ for sensitivity and specificity calculations.

## Discussion

This study is the first to show how NSCLC PD-L1 scoring using free open source software compares with the range of manual scores generated by a group of pathologists: QuPath automated PD-L1 scores showed agreement with average manual scores equivalent to that of individual pathologists, such that the overall accuracy of automated scoring could be considered comparable to that of individual pathologists. Values for agreement between pathologists in our study (1% threshold: 88% concordance, κ = 0.76; 50% threshold: 93% concordance, κ = 0.82) were similar to those in prior studies (e.g. 1% threshold: 84% concordance, κ = 0.54–0.63; 50% threshold: 82% concordance, κ = 0.75–0.83) [6, 14, 15]. We therefore considered the range of manual scores in our study to be an adequate representation of the variability typically seen in practice.

We also provide the first detailed step-by-step protocol with optimized settings for NSCLC PD-L1 scoring using a free open source platform. QuPath has a point-and-click style interface operable by any user, and may be implemented without delays for licensure or funding. To our knowledge, the only prior assessments of QuPath scoring in NSCLC compared to consensus pathologist scores did not report sufficient methodological detail for their classifier to be reproduced [12, 16]. Supporting the generalizable utility of QuPath, they reported a correlation with manual scores (R = 0.91) [12] similar to that in our study (concordance correlation coefficient = 0.925).

Consideration of the limitations of automated scoring identified in our study may play a role in the shaping the clinical use of automated scoring. Automated scores may be used as ‘tie breakers’ for difficult cases near cut offs, analogous to the use of immunohistochemistry to favor a diagnosis when morphology is ambiguous. QuPath may be of most use in confirming <1% and ≥50% scores, as automated scores in these categories were least likely to be discordant. One may have particular confidence in QuPath scores <1% or ≥50%, but put less weight on QuPath scores of 1–49%. Alternatively, if an automated system was used for the initial scoring of all cases, cases just above the 1% threshold and just below the 50% threshold may benefit from reflex manual review, as such cases are most likely to have discordant automated and manual scores.

Automated scoring may also have a use in quality assurance, serving as a consistent benchmark against which manual pathologist’s scores could be compared. Through comparison with automated scores, pathologists could compare their scoring to that of other pathologists without having to score the same cases (e.g. if pathologist 1 tends to score below automated scores and pathologist 2 tends to score above automated scores, pathologist 1 likely tends to score lower than pathologist 2). Comparison with automated scoring could also assess for drift in scoring tendencies over time, helping to identify when refresher training may be of value.

A time saving benefit of automated scoring may be realized in sites with an established workflow for slide scanning. The largest time investment in developing a QuPath scoring method was manual determination of an optimal threshold for staining intensity. We highlight this as one drawback of QuPath compared to approaches that use machine learning to identify an ideal threshold in an automated fashion. Before implementation in other laboratories or use with other PD-L1 staining protocols, the staining intensity threshold is likely to require re-optimization, as staining intensity may differ.

Training of a tumor vs. background classifier in QuPath required relatively few annotated cases: our classifier produced excellent results despite being trained on only 30 images from 10 cases. Interestingly, accuracy was similar for adenocarcinoma and squamous cell carcinoma test samples despite only three of the training images being from squamous cell carcinoma. A prior study using QuPath to score breast cancer biomarkers also had robust results despite training on only three cases per marker [10]. A custom classifier of similar accuracy may therefore be trained for local slides (to account for local differences in staining or include additional tumor morphologies) with minimal time spent annotating tumor areas and only a small number of cases needed. As alveolar macrophages misidentified as tumor cells contributed to falsely elevated automated scores, it may be particularly useful to include training set images with abundant alveolar macrophages that are manually annotated as such. Performance may also be improved by training on a greater number of cases capturing more variability in morphology.

However, significant improvement in the accuracy of automated scoring may be limited by the difficulty of balancing accuracy at two different thresholds. For instance, lowering the threshold for what intensity of brown coloration is called positive staining may improve accuracy at the 50% threshold by raising proportion of ≥50% calls, but may decrease accuracy at the 1% threshold by introducing false positive 1–49% calls. Similarly, optimization of cell size estimation (which determines whether membrane staining is detected as part of the cell) is limited by the variability of tumor cell sizes between cases. Increasing the “allowable” cell size may increase detection of positively staining large cells, but may incorrectly attribute the staining of inflammatory cells to adjacent small tumor cells. It’s possible that accuracy may be improved by developing multiple different algorithms customized for different ranges of cell size or PD-L1 staining (e.g., having small, medium and large cell versions of the algorithm, or using different thresholds depending on whether the case is anticipated to have high or low levels of PD-L1 staining); these possibilities remain to be explored. While the small size of most artifactual debris makes it difficult to annotate accurately, manual or automated artifact exclusion may also be explored.

We note that mucinous and sarcomatoid carcinomas were not included in our study as adequate case numbers were not available, and thus results may not be generalizable to these groups. Additional training and testing on small biopsy specimens is also advised prior to implementation on such specimens, as a greater degree of crush artifact and tissue fragmentation in small biopsies may reduce the accuracy of tumor cell recognition. Although the slides in our study were stained in several batches and therefore reflect some degree of intra-laboratory variation in staining, additional examination of how batch-to-batch variation in staining may affected QuPath scoring accuracy is warranted. We caution that the random trees classifier method used by QuPath is prone to overfitting, necessitating testing on additional independent validation sets prior to clinical use. Demonstration that automated and manual scoring are truly clinically interchangeable requires cohorts with treatment response data. Comparisons with outcome are beyond the scope of our study as the majority of cases in our study did not receive immunotherapy.

The present study demonstrates that automated PD-L1 scoring of NSCLC samples has an overall accuracy similar to that of individual pathologists, but has a tendency to predictably under- or over-estimate scores in particular scenarios. QuPath may be readily implemented following our step-by-step protocol, providing automated scores that may be of value in clinical PD-L1 testing workflows.

## Data Availability

The raw data supporting the conclusion of this article will be made available by the authors upon request, without undue reservation.
