# Supplementary material for: Automated PD-L1 Scoring for Non-Small Cell Lung Carcinoma Using Open-Source Software
Source: Pathol Oncol Res. 2021 Mar 26;27:609717. doi: 10.3389/pore.2021.609717 (PMC8262183; doi:10.3389/pore.2021.609717)
Supplement: Supplementary file 1 [file DataSheet1.PDF]

Supplemental Methods

Locally optimized parameters for NSCLC QuPath PD-L1 classifier generation and application are as follows:

Classifier training:

1. Open an image in QuPath (a scanned slide or tiff image). Stain recognition is performed per default settings. These can be confirmed by checking in the 'image' tab of the menu on the left side of the screen: Image type should be 'Brightfield H-DAB'. Stain 1 should be 'Hematoxylin: 0.651 0.701 0.29' and stain 2 should be 'DAB: 0.269 0.568 0.778'.
2. Draw a box around the area to be scored using the rectangle tool.
3. Select 'Cell detection' from the 'Analyze, Cell analysis' menu
4. Set parameters as follows:
    a. Adjust the following parameters:
        i. Cell expansion: 4.4463 µm
        ii. Requested pixel size: 0.25 µm
        iii. Unselect 'exclude DAB (membrane staining)'
    b. The remaining parameters are left as default:
        i. Detection image: hematoxylin OD
        ii. Background radius 8 µm
        iii. Median filter radius: 0 µm
        iv. Sigma:1.5 µm
        v. Minimum area 10 μm^2
        vi. Maximum area: 400 μm^2
        vii. Intensity threshold: 0.1
        viii. Max background intensity: 2
        ix. Check 'split by shape'
        x. Check 'include cell nucleus'
        xi. Check 'smooth boundaries'
        xii. Check 'make measurements'
5. In the 'Analyse, Calculate features' menu, select 'Add smoothed features'
    a. Set radius to 50 µm and leave unchecked 'smooth within classes' and 'use legacy name features'
    b. Press 'run'
6. Annotate tumor and background areas:
    a. Using the polygon tool, draw an annotation around a tumor area. Right-click and in 'select class' choose 'tumor'
    b. Using the polygon tool, draw an annotation around a non-tumor area. Right-click and in 'select class' choose 'stroma'
    c. Repeat 'a' and 'b' above on several tumor and non-tumor areas
7. In the 'Classify' menu, select 'Create detection classifier'
    a. Leave classifier type as the default 'Random trees'
    b. For 'Intensity feature' select 'Cell: DAB OD mean'
    c. Check 'Use single classifier' and set threshold to 0.06 **(Note that this parameter is likely to need re-optimization for locally stained slides, given that staining intensity may differ between laboratories and staining protocols)**

    d. Select ‘Build and apply’. The classifier mark-up should be shown on the tissue image (default colors: negative tumor blue, positive tumor red, stroma green). The classifier will by default use all available features (listed below).
    e. Leave the classifier window open to allow refinement in the steps below
8. Refine classifier performance
    a. On the currently open slide, you can annotate additional tumor and stroma areas, and the classifier mark-up should auto-update (or you can press the auto-update button to show the revised classifier).
    b. Open a new image and select ‘Yes’ to ‘Retain current training objects in classifier?’
    c. Repeat steps 1-6. The classifier mark-up should auto-update (or you can press the auto-update button to show the revised classifier).
        i. You can check you’ve included in training the annotations on prior images by clicking in the create classifier window ‘advanced options, more, show training object counts’
    d. Continue training on additional images until satisfied with the performance of the classifier.
    e. Save the classifier using the ‘Save classifier’ button at the bottom of the classifier window. Note that the classifier cannot be further trained once saved.

Implementation of scoring using a trained classifier:

1. Perform steps 1 through 5 above.
2. In the ‘Classifier, Load classifier’ menu, press the ‘Load classifier’ button, select the saved classifier file and click ‘Open’. It will automatically run (allow several seconds for results to appear).
3. To see results, click on the annotations tab in the menu at the left side of the screen. The ‘Tumor: Positive %’ provides the digital PD-L1 score.

Features used in classifier training:

Nucleus: Area
Nucleus: Perimeter
Nucleus: Circularity
Nucleus: Max caliper
Nucleus: Min caliper
Nucleus: Eccentricity
Nucleus: Hematoxylin OD mean
Nucleus: Hematoxylin OD sum
Nucleus: Hematoxylin OD std dev
Nucleus: Hematoxylin OD max
Nucleus: Hematoxylin OD min
Nucleus: Hematoxylin OD range
Nucleus: DAB OD mean
Nucleus: DAB OD sum
Nucleus: DAB OD std dev
Nucleus: DAB OD max
Nucleus: DAB OD min
Nucleus: DAB OD range
Cell: Area
Cell: Perimeter
Cell: Circularity
Cell: Max caliper
Cell: Min caliper
Cell: Eccentricity
Cell: DAB OD mean
Cell: DAB OD std dev
Cell: DAB OD max
Cell: DAB OD min
Cytoplasm: DAB OD mean
Cytoplasm: DAB OD std dev
Cytoplasm: DAB OD max
Cytoplasm: DAB OD min
Nucleus/Cell area ratio
Smoothed: 50 µm: Nucleus: Area
Smoothed: 50 µm: Nucleus: Perimeter
Smoothed: 50 µm: Nucleus: Circularity
Smoothed: 50 µm: Nucleus: Max caliper
Smoothed: 50 µm: Nucleus: Min caliper
Smoothed: 50 µm: Nucleus: Eccentricity
Smoothed: 50 µm: Nucleus: Hematoxylin OD mean

Smoothed: 50 µm: Nucleus: Hematoxylin OD sum
Smoothed: 50 µm: Nucleus: Hematoxylin OD std dev
Smoothed: 50 µm: Nucleus: Hematoxylin OD max
Smoothed: 50 µm: Nucleus: Hematoxylin OD min
Smoothed: 50 µm: Nucleus: Hematoxylin OD range
Smoothed: 50 µm: Nucleus: DAB OD mean
Smoothed: 50 µm: Nucleus: DAB OD sum
Smoothed: 50 µm: Nucleus: DAB OD std dev
Smoothed: 50 µm: Nucleus: DAB OD max
Smoothed: 50 µm: Nucleus: DAB OD min
Smoothed: 50 µm: Nucleus: DAB OD range
Smoothed: 50 µm: Cell: Area
Smoothed: 50 µm: Cell: Perimeter
Smoothed: 50 µm: Cell: Circularity
Smoothed: 50 µm: Cell: Max caliper
Smoothed: 50 µm: Cell: Min caliper
Smoothed: 50 µm: Cell: Eccentricity
Smoothed: 50 µm: Cell: DAB OD mean
Smoothed: 50 µm: Cell: DAB OD std dev
Smoothed: 50 µm: Cell: DAB OD max
Smoothed: 50 µm: Cell: DAB OD min
Smoothed: 50 µm: Cytoplasm: DAB OD mean
Smoothed: 50 µm: Cytoplasm: DAB OD std dev
Smoothed: 50 µm: Cytoplasm: DAB OD max
Smoothed: 50 µm: Cytoplasm: DAB OD min
Smoothed: 50 µm: Nucleus/Cell area ratio
Smoothed: 50 µm: Nearby detection counts
